# The Pandora’s box of transparency opened by direct-to-consumer genetic testing

**DOI:** 10.3389/fgene.2023.1175666

**Published:** 2023-05-19

**Authors:** Garbiñe Saruwatari Zavala

**Affiliations:** Department of Ethical, Legal and Social Studies of the National Institute of Genomic Medicine-Ministry of Health (Instituto Nacional de Medicina Genómica-Secretaría de Salud), Mexico City, Mexico

**Keywords:** assisted reproductive technology, donor conceived, fertility fraud, DTC genetic testing, right to know, genetic identity

## Abstract

With the increasing use of direct-to-consumer (DTC) genetic testing, several cases of fertility fraud have been uncovered throughout the world. A review of newspaper articles and specialized literature was made to analyze the issue of fertility fraud. The most famous cases, some of which are narrated in this article, became a scandal because they were committed by the doctors who treated the patients in question as a routine procedure in their medical practice. Some have been widely exposed in documentaries on streaming platforms, thereby raising awareness about a grave problem. The discussion focuses on the ambiguous regulation on the anonymity of donors, which has been one of the elements of the deception committed against the families using these services. Anonymity nowadays collides with the fascinating Pandora’s box of transparency in genetic information that has been opened by DTC genetic testing.

## 1 Introduction

In the 1970s, infertility was a relatively new medical specialty, and there were no big sperm banks yet. Although these banks started emerging in the 1980s, standardization tests were barely being done to guarantee the optimal state of the biological material over long periods of time. Moreover, catalogs of donors to select by criteria such as eye color or hobby were not available in all clinics. Doctors usually found the donors themselves, often among medical trainees, who had the advantage of being readily available to provide fresh sperm when needed while having a reputation for being successful young men (Zhang, 2019a). In this mid-late 20th century context, medical staff usually advised parents not to tell their children how they were conceived and to extend the secrecy to other members of the family. This was in part due to the uncertainty over who would be the legal father of a donor-conceived child but also because of the social concept of family bonds.

This article narrates several cases in which doctors impregnated their patients with their own sperm. They lied about the origin of the biological material telling their patients it was obtained from an anonymous donor. These doctors believed that they had covered their crimes and gotten away from legal accountability because no one knew how they proceeded. Nevertheless, the years passed, and DTC genetic testing revealed a shocking truth for both the patients and the donor-conceived people. Through DTC testing platforms, such as 23andMe and Ancestry.com, many users noticed that they were related to each other and later found out that their father was the fertility specialist who their parents had consulted three decades earlier.

Half-siblings conceived by these fraudulent physicians began to connect with each other, and the lurid details about the doctors’ actions were exposed on the media. With the streaming of Lucie Jourdan’s documentary *Our Father* on Netflix, many viewers learned about the case of Donald Cline. It tells the real-life story of former fertility doctor Donald Cline, who used his own sperm throughout his career to impregnate unsuspecting patients. (Jourdan, 2022).

Unfortunately, this was neither the first time nor the only case in which alleged specialists or even renowned experts in assisted reproduction have committed fraud and other crimes. Among them, the cases of Paul B. Jones, John Boyd Coates III, Cecil Jacobson, Quincy Fortier, Jos Beek, and Jan Kaarbat are all well documented by journalists, and now by their conceived children.

In addition to the serious ethical and legal implications concerning the lack of control of certain assisted-reproduction centers and the legal loopholes in several countries on the subject, the Cline case opens several aspects to be considered in the debate on direct-to-consumer genetic tests (DTC tests). Some of these aspects are the informed consent of consumers, incidental findings in tests, the right to know about one’s own genetic origin, and the usefulness of tests for several other purposes, such as identification of unknown corpses and for criminal matters.

## 2 Subsections relevant to the topic

### 2.1 Insemination fraud

#### 2.1.1 Donald Cline

Liz White and her husband tried to conceive for two and a half years; they had seen a doctor who attempted insemination with frozen donor sperm, which was a new technique at the time that was not successful for them. Someone recommended Dr. Cline to them. He was a top fertility doctor that opened his clinic in 1979 in Indianapolis. He had many patients and used fresh sperm, which had higher success rates back then. Cline told them that he would use the sperm of a medical resident whose appearance and blood type matched with White’s husband’s and that he only used each donor for three successful pregnancies (Zhang, 2019b).

Jacoba Ballard grew up suspecting that she was adopted because she had no physical resemblance to the rest of her family. When she was 10 years old, her parents told her that they had used donated semen to conceive her. In 2014, after taking a 23andMe ancestry test, she discovered she had seven half-siblings; she then contacted them to learn about their mysterious family connection, only to learn that each of the mothers had seen the same fertility specialist. No one in the 23andMe database shared enough DNA with them to be their father, but they found dozens of more distant genetic matches. By combing through public records and social-media profiles and sometimes simply asking their genetic matches about their families, they could build a giant family tree that, they hoped, would eventually lead to their father. Finally, a woman who shared some of their DNA told them she had a cousin named Donald Cline, a doctor in Indianapolis. Some of the half-siblings met with Cline, who admitted using his own sperm but said the records had been destroyed years before.

When they were able to match the information, Cline’s progeny filed their complaints with the Indiana Attorney General, who sent Cline a letter describing the allegations against him. He responded back at least twice saying he had never used his own sperm and that any woman who had said otherwise should be considered guilty of slander and/or libel. In September 2016, the Marion County Prosecutor charged Cline with two counts of felony obstruction of justice as he was discovered lying to investigators about whether he inseminated patients using his own sperm. Investigators showed up at Cline’s house with a search warrant to acquire DNA material from him; after swabbing his mouth, the truth came into light. Cline was ultimately fined $500 and received a 1-year jail sentence. However, he was later given a year of probation, and he also voluntarily surrendered his medical license on 23 August 2018, despite having been retired since 2009 (Madeira, 2019).

Several of the supposed donor-conceived individuals and their mothers have also filed civil lawsuits against Cline for fertility fraud, but he was not charged with anything related to it since Cline’s actions were not illegal in Indiana at the time they were committed.

#### 2.1.2 Paul B. Jones

In the late 1970s, Cheryl Emmons and her husband, John, who had had testicular cancer, saw Dr. Paul B. Jones at Women’s Healthcare of Western Colorado. The doctor promised that he would use donated sperm from some medical student who looked like John. Their first daughter, Maia Emmons-Boring, was born in 1979, and their second daughter, Tahnee Scott, came in 1984. They were both conceived by artificial insemination. Dr. Jones was also the gynecologist in charge of her deliveries.

In 2018, Maia took a DNA test to find out her ancestry through Ancestry.com and found that she had several half-siblings. They got in touch and realized that they had in common that their mothers had gone to the same fertility specialist, who used his own sperm to inseminate his patients. Maia also confirmed this information when she saw the great physical resemblance she had to the doctor (Tabachnik, 2019).

When Emmons and her daughters sued Jones in October 2019, they were not the only ones—a total of seven families sued the physician. That said, it has been found that at least a dozen women have been inseminated in the same manner for more than two decades. Jones resigned his medical license the month after the lawsuit filing. Before trial, five of the families settled for an unknown amount, while the other two followed suit. The Emmonses filed more charges against Jones than against the clinic hoping that most of the penalty would be paid by him. On 27 April 2020, a Mesa County District Court jury awarded the plaintiffs $8.75 million (Lukpat, 2022).

Betty and Karl Stephens, along with their daughter Nichole Long, also filed a lawsuit against Dr. Jones, the clinic, and Dr. Stephen Meacham, who worked with Jones over a period of 20 years, so he knew that he was using his own semen and might have even helped him. In this lawsuit, twice as many patients were added than in the previous one (Tabachnik, 2021). In addition, the family claims that Jones was a carrier of cystic fibrosis and that several of Nichole’s half-siblings also inherited the genetic disease, which has various health complications, including persistent and recurrent lung infections.

The lawsuits allege medical malpractice, lack of informed consent, fraud, misrepresentation, breach of contract, assault, extreme and outrageous conduct, and breach of fiduciary duty added in the second complaint. This case prompted Colorado legislators to take action to make insemination with the doctor’s own sperm without the patient’s consent a felony.

#### 2.1.3 John Boyd Coates III

In 1977, Cheryl Rousseau went to Dr. John Boyd Coates III requesting to be inseminated with donated sperm after discovering that her husband’s vasectomy was irreversible. Forty years later, her daughter, Barbara, began searching for her biological father by taking a genetic test and comparing the sites Ancestry.com and 23andMe to find him. She was shocked when the results led back to Coates, realizing that he was her biological father. At the consultation, Dr. Coates had lied to Cheryl saying that the sperm donor would be an anonymous medical student with similar traits to her husband’s (Dyer, 2022).

Cheryl and her husband, Peter Rousseau, sued Coates in late 2018, and the civil case was heard in federal court in Burlington on charges of breach of contract, assault, and fraud. The focus of the trial was not to determine whether Coates used his own sperm to impregnate Rousseau but rather to determine the damages and relief she should receive for the deception.

Another patient similarly sued Coates, with comparable facts and claims. Her daughter published an ad in the newspaper to find other siblings, stating that whoever had been born by assisted reproduction provided to their parents at the Associates of Gynecology and Obstetrics clinic in Berlin, Vermont, or the Central Vermont Hospital between the years 1974–1986, or at Dr. Coates’ office at Mountain View Physicians in Burlington, Vermont, between 1986 and 2009, should contact her to find if they had a genetic match with Coates (Keays, 2022a).

During the trial, Coates said that the court order to perform a genetic test seemed like a great invasion of his privacy and his lawyer added that Coates’ intention was never to harm but rather help his patients. The Vermont Board of Medical Practice revoked Coates’ medical license for serious violations of the law and trying to mislead investigators. On 30 March 2022, the Court awarded Cheryl Rousseau $250,000 in compensatory damages and $5 million in punitive damages, crediting all charges in the lawsuit (Keays, 2022b).

#### 2.1.4 Cecil Bryant Jacobson

Jacobson operated a reproductive genetics center in Fairfax County, Virginia, in the 1980s. He treated several patients who had problems getting pregnant or carrying a pregnancy to term by injecting them with the hCG hormone before and after conception. In the early stages, the pregnancies seemed to progress normally, the women had the expected bodily changes associated with pregnancy, and the tests were positive. Some women did become pregnant and gave birth while others did not. In the latter’s case, around the third month of pregnancy, Jacobson told them that the fetus had died.

Years later, a mother noticed the great physical resemblance between her son and the doctor who treated her years before. Likewise, other women who had been his patients became suspicious of Dr. Jacobson and gave the information to a local television station, which conducted the journalistic investigation on the false-pregnancy cases (Roig, 1992).

The patients sued Jacobson on charges of fraud and perjury for giving false testimony during the civil proceeding. It was discovered that the women had positive results and body changes because of the hormone. They were never actually pregnant although Jacobson made them believe they were and showed them ultrasounds in which the fetus was supposedly identified. Later during the trial, experts proved that the ultrasound images of the alleged fetuses were either nearby organs or fecal matter.

Another type of fraud was also discovered—Jacobson inseminated with his own sperm patients who believed they were being inseminated with gametes from an anonymous donor program or with their partner’s sperm. He claimed that he only used his own sperm when donors did not show up for appointments or to protect his patients from HIV/AIDS. Three receptionists and a lab technician who worked there testified nevertheless that there were never any anonymous donors at the clinic. This is in addition to the fact that the researchers did not find any databases with the identities of the donors, which Jacobson claimed to have. Various people underwent a genetic test and found 15 children linked to Jacobson, but he is suspected of having been the biological father of at least 75 people.

In 1992, Jacobson was sentenced to 5 years in prison on 52 counts of mail fraud and perjury, and a fine of $116,805, in addition to having his medical license revoked (NYT, 1992). In 1994, a federal judge ruled that Jacobson could no longer remain free on bond since all his appeals were exhausted, so he reported to a federal prison in Florence, Colorado, in 1994 to begin serving his sentence (Miller, 1994).

#### 2.1.5 Quincy Fortier

Dr. Quincy Fortier was a Las Vegas, Nevada, fertility specialist for over 60 years. His patients and colleagues trusted him, so much so that he was even named “Doctor of the Year” in 1991 by the Clark County Medical Society, according to the Las Vegas Review-Journal.

Wendi Babst, a recently retired detective who had taken up genealogy, discovered through a 23andMe test that Fortier was her father and began to investigate him on her own. The film director Hannah Olson contacted her for the making of the documentary *Baby God*, broadcasted on HBO in December 2020. (Olson, 2020). In her personal search, Babst, along with Olson, discovered various women who Fortier had inseminated with his own sperm without their consent. This happened not only to women who came looking for an anonymous donor but also to couples who believed that their own sperm was being used in the procedure. Fortier’s crimes go further, however, because he also inseminated patients who came to the clinic for a routine check-up and did not want to get pregnant (Borden, 2020).

It was also known that he impregnated his own stepdaughter without her consent and imprisoned her in a house for single mothers during the pregnancy. She gave her baby up for adoption (Lorusso, 2020). Likewise, Fortier sexually abused his other daughters and son, who he had during his marriage.

Several patients sued Fortier, but he was acquitted since there were no laws in the state of Nevada against such acts. He never revealed how many patients he had treated and died in 2006 at the age of 94 without being convicted.

#### 2.1.6 Jos Beek

Gynecologist Jos Beek, who for 15 years practiced at the Sint Elizabeth Hospital in Leiderdorp, today Alrijne Hospital, in the city of Leiden, the Netherlands, was found to have secretly used his own sperm to treat his patients, even though they had asked to be inseminated with sperm from an anonymous donor.

A spokesperson for the Alrijne Hospital explained that, in June 2021, Fiom, an organization specializing in paternity issues, approached the hospital authorities on behalf of 21 people seeking identification of Beek as their father, which was verified by a DNA test. The hospital authorities summoned possible affected individuals, and it was determined through an independent committee that the figure amounts to at least 41 children. The eldest (known) son was born in 1975 while the youngest came in 1990. Beek died in 2019, so he could not be tried for his actions (Boffey, 2022).

In addition, the hospital revealed that the gynecologist was also found to be a carrier of a rare hereditary condition. Two of the conceived children, who were born to the same mother, died during infancy, thus both the mother and Beek were found to be carriers of the same genetic condition (DN, 2022).

#### 2.1.7 Jan Kaarbat

Dr. Kaarbat was a fertility specialist who ran the clinic in the Barendrecht municipality in Rotterdam, the Netherlands. Before the trials to request the comparison of his DNA, he already had a history of suspicious behavior. In 2009, he was forced to close the clinic when the health inspection observed “serious administrative problems, with a lack of adequate records and documentary disorder” (Ferrer, 2019).

It was not until 2017 that a group of people requested in court that Dr. Kaarbat take a DNA test on the suspicion that he had used his own sperm to inseminate various women at the Blijdorp clinic. He denied the accusations and refused to cooperate and undergo a genetic test, claiming an invasion of his and his family’s privacy, fearing there would be inheritance-related issues. He died in April 2017, a month before the start of the trial; nevertheless, the court ordered that the genetic material found in 27 objects of his property be preserved to carry out the comparison with the alleged children. Moreover, one of the recognized sons of the doctor agreed to take a test that same year to compare his DNA with that of the son of a patient treated by Kaarbat.

In February 2019, the court authorized that the interested parties could compare their DNA with that of the doctor through the personal objects that were kept of his. The analyzes were carried out at the Canisius Wilhemina hospital in the city of Nijmegen. In April of the same year, the test results confirmed the suspicions. In total, it is estimated that Kaarbat had at least 71 children: 22 with his three wives and other partners, plus 49 cases of children born by artificial insemination with non-consensual biological material. New cases could appear since, in the 1980s and 1990s, some 6,000 women passed through his fertility clinic, where about 10,000 children were conceived, according to the doctor himself (Harris, 2019). It is feared that approximately 200 children could be his.

Several people have continued to claim similar cases to be able to compare their DNA, with the support of the NGO Defence for Children. It is to be decided whether to claim compensation from Karbaat’s family for the expenses caused by the process and for the emotional damage suffered (Caldwell, 2022).

In the documentary *Seeds of Deceit*, directed by Miriam Guttmann and released in 2018, some of the biological children of Karbaat meet for the first time and share their conflicted feelings about finding out their father isn’t who they thought he was (Guttmann, 2018).

### 2.2 Legislation ambiguity

A point in common between the seven doctors is that they all committed these acts routinely—it was not an isolated or accidental act; each one used their sperm dozens of times to the point that many of them have not been able to state exactly how many children they fathered. For instance, Karbaat is presumed to have had about 60 children with the women he claimed to have helped (Richardson, 2020). Even now, it remains unclear how many children are biologically his. We could call them *serial inseminators* since, as in other cases of rape and abuse, desires for control, the exercise of power over the victim, and narcissistic personality traits are involved. It should be added that, in the 1970s and 1980s, as it was previously mentioned, fresh semen was used since its conservation through cryopreservation had not yet been perfected. This leads to the victims imagining, with horror, how these doctors left them lying down during the consultation, went to the continuous room to masturbate, returned to the examination room, and immediately impregnated them. Obtaining the biological sample from an anonymous donor who does not know and has never met the woman is not the same as getting it from the doctor himself, who is treating and touching the patient, masturbating on the spot (Ettachfini, 2019).

Cline’s case falls between civil and criminal law. His conduct was not classified as rape or sexual assault because, under Indiana law, rape only occurs when “a person knowingly or intentionally engages in sexual intercourse or sexual conduct with another person who is compelled by force or imminent threat of force, unaware that the sexual conduct is occurring, or is incompetent and cannot consent to sexual conduct.” It is difficult to prove that Cline’s actions were sexually motivated without an admission from him saying so, and it appears that women consented to the insemination when they were told about the anonymous semen donor (Madeira, 2020).

In addition to the above, Jacobson refined his base act by not only using his sperm and lying about its origin but also providing hormones to delude patients with false pregnancies. In turn, Fortier inseminated patients who did not want to get pregnant and sexually abused his own children. It is also striking how Cline’s children, in the documentary, reflect on the fact that one of the reasons the doctor might have had is the desire to spread his genetic information under the notion of his supposed racial superiority and belief in his superior intelligence. Several of them surely had a similar motivation since, in the cases of Jones and Beek, they still used their own sperm knowing they were carriers of a genetic disease.

Currently, the recognition of fertility fraud has increased both in law (Bice, 2022) and verdicts. On 6 January 2023, the Superior Court of Pennsylvania dismissed a case based on lack of jurisdiction that shows that this type of fraud can be committed not only by experts in reproductive medicine but also by the parents themselves (Supreme Court of Appeals of the State of West Virginia, 2021). It is about the custody claim by an intersex father (identified in the trial as “Father”) who claims to be father and mother at the same time to his three children. Although he was registered as a woman on his birth certificate, the Father points out that he always considered himself a man. Before undergoing some corrective surgeries not specified in the trial, he decided to freeze his ova before meeting his partner.

The Mother and Father had a child by artificial insemination performed by the Father, a registered nurse. The Mother thought that the Father’s own sperm was being used, which was not the case because he could not provide the sperm; during the trial, he refused to reveal the source. For the next three children, they resorted to *in vitro* fertilization, in which the Mother believed that her own eggs and the Father’s sperm would be used, carrying out the gestation herself. In the case of the four children, he was registered on the birth certificates as the father and she as the mother. During the custody trial, the Mother found out that she carried the three pregnancies to term using the Father’s ova without having ever been told about it by him. While the Father argues that the Mother is only a gestational carrier and should therefore not have custody rights, she lives in fear that, in the various lawsuits still pending, her rights will not be recognized due to not having any genetic affiliation with the children (Trachman, 2023). In this type of cases, the focus should be not only on the woman deceived through fraud and lack of consent but also on the affectation towards the children. While the Father argues based on genetics, the Courts that shall decide the various trials in progress should take into consideration who is the best legal guardian for the socio-affective development of the children in accordance with the minors’ best interests.

### 2.3 Comparative law on anonymity

Riaño and contributors (Riaño-Galán et al., 2021) highlight the difference between the right to privacy and confidentiality of submitting to assisted reproductive techniques, and the anonymity of gamete donors. Mulligan (2022) adds a third matter on which to reflect: the anonymous birth.

In the first scenario, the right of patients to privacy is an inalienable right with the correlative duty to confidentiality on the part of health personnel. This right considers that patients may or may not communicate to their children that they have been users of assisted-reproduction services. The parents may choose whether anonymity or identifiability is best for their child and the family (Ravitsky, 2017).

In the scenario of donor anonymity, the usual practice in many countries, whether they had specific regulations on the matter or not, was maintaining such anonymity, with certain exceptions in which to reveal their identity, like having a serious illness that endangered the life or health of the resulting children and/or the donor. In many cases, there was no direct contact between the donor and the resulting child but rather between health institutions, for example, between the assisted-reproduction clinic and the hospital where the person who discovered the disease was being treated (either the child or the parent).

In the third scenario, when giving birth, the mother wants to keep her identity a secret so that her family does not find out or because she wants to give the child up for adoption. Mulligan explains the public-health (abandonment of the newborn when it is compulsory to register the mother’s name in a certificate) and social reasons (rape committed against the mother) for which, in some countries, the practice of anonymity of the mother has been regulated or tolerated so as not to generate filiation with the child. Nevertheless, in this scenario, the arguments for anonymity do not start from the same premises as those in the case of gamete donors.

As stated, some countries accept as an exception to the general rule of anonymity, revealing information only when a serious hereditary disease is found. Twenty years ago, the Dutch press exposed the case of a man who had donated sperm in the late 1980s, at the Jeroen Bosch Hospital in Den Bosch (Gebhardt, 2002). After 6 years donating sperm, the man was diagnosed with autosomal dominant cerebellar ataxia (ADCA), a severely disabling neurological disease, which does not occur before puberty and may remain latent for many years. The sperm was used for the conception of 18 children in 13 women. The parents were informed 3 years after the insemination, whilst the hospital obtained expert opinions and the families were located, since due to the privacy laws in force at the time, it was impossible to use some of the address records.

Although this is not a recent case, at that moment it raised three main concerns that are still relevant. At that time genetic testing did not detect ADCA. Even years later, Isley et al. (2016) point out that risks cannot always be detected at the time of a donor eligibility assessment due to reduced penetrance, variable expressivity, or temporal factors. They even recommend various additional actions to genetic tests, in order to measure genetic risk. Gamete donors should be screened for recessive conditions such as cystic fibrosis, thalassaemia, Tay Sachs disease, Gaucher’s disease, spinal muscular atrophy, sickle cell anemia, as well as other so-called “rare” diseases (NIH-GARD, 2023).

Another consideration, related to the first scenario proposed by Riaño, is that by the time the parents were informed, not all of them had revealed to their children that they were donor-conceived. If the media that broadcasted the story had exposed the situation before the families were contacted, it would have caused great anxiety in the couples (Delaytycki, 2002) and maybe breached the family privacy. Additionally Guido de Wert ponders the child’s right not to be informed of a genetic disease that has no preventive or therapeutic options (Gebhardt); at an age when the child can make an informed decision or, in adulthood can decide to get tested and make an informed decision about reproduction (Sheldon, 2002).

To recap, when it comes to gamete donation, the argument reiterated by clinics and specialists who want anonymity to remain is that the right to privacy might be violated, coupled with the fact that donors would be discouraged because they do not want to have problems later in life regarding the issues of paternity and responsibility. By the other side, the reason given by the countries that have regulated the specific point regarding revealing donor’s information is the right to identity. This change has been driven by the activism of donor-conceived people who feel wronged by their inability to access information on their genetic origins (Table 1).

**TABLE 1:** Comparative law on anonimity.

	Europe & Oceania	America	Asia & Africa
NO ANONYMITY	Australia: Narelle’s Law of the state of Victoria		
	Austria: Reproductive Medicine Act		
	Finland: Act on Assisted Fertility Treatments 1237/2006		
	France: Public Health Code and Law n. 2021–1017 relating to Bioethics amended by Decree n. 2022–1187		
	Germany: Sperm Donor Registry Act		
	Ireland: Children and Family Relationships Act		
	Netherlands: Embryo Law and Law on data from donors for artificial reproduction		
	New Zealand: Human Assisted Reproductive Technology Act		
	Norway: Norwegian Act on Artificial Fertilization No. 68/1987 and Act on the Medical Use of Biotechnology		
	Portugal: Law No. 32/2006 Concerning Medically Assisted and Decision of the Constitutional Court No. 225/2018		
	Sweden: Swedish Insemination Act		
	Switzerland: Reproduction Medicine Act		
	United Kingdom: Human Fertilisation and Embryology Act and The Human Fertilisation and Embryology Authority Regulations		
HYBRID MODEL	Belgium: Law No. 6/7/2007 on Medically Assisted Reproduction and the Disposition of Supernumerary Embryos and Gametes	United States: The Fertility Clinic Success Rate and Certification Act	
	Denmark: Act No. 460/1997 on Artificial Fertilization		
	Iceland: Acts No. 55/1996 and 54/2008 on Artificial Fertilisation and use of Human Gametes and Embryos for Stem-Cell Research		
ANONYMITY	Estonia: Artificial Insemination and Embryo Protection Act	Canada: Assisted Human Reproduction Act	Japan: Act No. 76 of 202 Law on special provisions of the Civil Code concerning the provision of assisted reproductive technology and the parent–child relationship of children born as a result
		Argentina: Law No. 26.862, Medically Assisted Reproduction on comprehensive access to medical assistance procedures and techniques for medically assisted reproduction; Civil and Commercial Code, approved by law 26,994, enacted by decree 1795/2014	Singapore: The Licensing Terms and Conditions on Assisted Reproduction Services; The Status of Children on Assisted Reproduction Technology Act
	Spain: Laws 35/1988, 45/2003 and 14/2006 on Assisted Human Reproduction Techniques	Colombia: Law No. 1953, by means of which the guidelines are established for the development of public policy for the prevention of infertility and its treatment within the parameters of reproductive health	South Africa: National Health Act No. 61/2003
		Uruguay: Law No. 19.167 Regulation of Assisted Human Reproduction Techniques	

#### 2.3.1 No anonymity

The regulation in some countries has moved (McDermott et al., 2022) from the protection of anonymity to its total prohibition or to the donor-conceived individual’s right to know their origin at a certain age.

• Australia: state of Victoria [1998]. In 2005, a national medical guideline stipulated the abolishment of anonymous donations in all states. In 2018, Victoria went even further in the so-called “Narelle’s Law,” granting all-donor conceived people the opportunity to receive identifying information about their sperm, oocyte or embryo donor retroactively for donations made before 1998 (Allan, 2016).
• Austria: Reproductive Medicine Act [1992], amended in 2015 (TFP, 2015).
• Finland: Act on Assisted Fertility Treatments 1237/2006, which entered into force in 2007 (Finnish Ministry of Justice, 2007).
• France: Public Health Code [1994] and Law n. 2021–1017 relating to Bioethics [2021] (Journal officiel Lois et Décrets, 2021), amended in 2022 by Decree n° 2022–1187 (Journal officiel Lois et Décrets, 2022).
• Germany: Sperm Donor Registry Act [2017] (TFP, 2018) entered into force in 2018 (Steiwer, 2017).
• Ireland: Children and Family Relationships Act, amended in 2015 (Department of Health, 2020).
• Netherlands: Embryo Law (Government of the Netherlands, 2002a) and Law on data from donors for artificial reproduction (Government of the Netherlands, 2002b) (Janssens et al., 2006).
• New Zealand: Human Assisted Reproductive Technology Act [2004], entered into force in 2005 (Parliamentary Counsel Office, 2004).
• Norway: Norwegian Act on Artificial Fertilization No. 68/1987 and Act on the Medical Use of Biotechnology [2003], amended in 2020 (Bjerke, 2020).
• Portugal: Law No. 32/2006 Concerning Medically Assisted Procreation (Assembleia de República, 2005) and Decision of the Constitutional Court where Portugal considers that anonymity is not constitutionally admissible, 24 April 2018, No. 225/2018 (De Sutter, 2018).
• Sweden: Swedish Insemination Act [1984], which entered into force in 1985 (Nordic Council of Ministers, 2006)
• Switzerland: Reproduction Medicine Act (Federal Office of Public Health, 2001).
• United Kingdom: Human Fertilisation and Embryology Act 1990 (Frith et al., 2007) and The Human Fertilisation and Embryology Authority [Disclosure of Donor Information] Regulations (Human Fertilisation and Embryology Authority, 2004).

#### 2.3.2 Hybrid model

In this model, donors can choose whether to donate anonymously or not.

• Belgium: Law No. 6/7/2007 on Medically Assisted Reproduction and the Disposition of Supernumerary Embryos and Gametes (De Neubourg et al., 2013).
• Denmark: Act No. 460/1997 on Artificial Fertilization (IVF Abroad, 2022).
• Iceland: Act No. 55/1996 on Artificial Fertilisation and use of Human Gametes and Embryos for Stem-Cell Research, amended by Act 54/2008 (Ministry of Welfare, 1996, Ministry of Welfare 2008).
• United States: The Fertility Clinic Success Rate and Certification Act (U.S. Congress, 1992) was introduced to legislate assisted human reproduction at a federal level; regulations vary at the individual state level.

#### 2.3.3 Anonymity

Some countries still maintain complete anonymity or provide for certain exceptions.

• Canada: Assisted Human Reproduction Act [2004] (Motluk, 2020).
• Estonia: Artificial Insemination and Embryo Protection Act [1997], amended in 2003 (State Chancellery, 2003).
• Japan: Act No. 76 of 202 Law on special provisions of the Civil Code concerning the provision of assisted reproductive technology and the parent–child relationship of children born as a result (Yamada et al., 2022).
• Singapore: The Licensing Terms and Conditions on Assisted Reproduction Services [2011], revised in 2020, (Ministry of Health, 2011, Ministry of Health 2020), is a set of administrative rules for clinics and personnel. The Status of Children on Assisted Reproduction Technology Act [2013] was amended in 2021 and entered into force in 2022 (Law Revision Commission, 2013, Law Revision Commission 2021).
• South Africa: National Health Act No. 61/2003 [2003], amended in 2016 (Wijnland Fertility, 2017).
• Spain: Law 35/1988 on Assisted Human Reproduction Techniques, amended by Law 45/2003 (Gobierno de España, 2003) and by Law 14/2006 (Gobierno de España, 2006).
• Uruguay: Law No. 19.167 Regulation of Assisted Human Reproduction Techniques (Ministerio de Salud Pública, 2013).

Spanish, Singaporean, Estonian and Uruguayan specific laws establish a general rule that gamete donation is provided on an anonymous basis, subject to some very limited exceptions and meeting certain requirements, such as having a serious disease or going through legal proceedings. As an example, Estonian law indicates a limited list of the types of information concerning the biological and social background of the donor that the recipient has the right to know.

In Latin America, Argentina and Colombia, which are the only countries that have specific laws regulating assisted reproductive techniques apart from Uruguay, do not say anything about anonymity. In Argentina, it is not the specific law, but rather the Civil and Commercial Code, that allows the exception of anonymity derived from a judicial process (Lima and Rossi, 2019).

• Argentina: Law No. 26.862, Medically Assisted Reproduction on comprehensive access to medical assistance procedures and techniques for medically assisted reproduction (Honorable Congreso de la Nación, 2013); Civil and Commercial Code, approved by law 26,994, enacted by decree 1795/2014.
• Colombia: Law No. 1953, by means of which the guidelines are established for the development of public policy for the prevention of infertility and its treatment within the parameters of reproductive health (Congreso de Colombia, 2019).

Mexico and several other Latin American countries do not even have a specific regulation on assisted reproduction; the donation of gametes follows the same criteria as the donation of other cells, tissues, and organs, so it is anonymous (Observatorio de Igualdad de Género de América Latina y el Caribe, 2021).

### 2.4 Confidentiality and anonymity v. right to identity

The series of the above-mentioned events lead us to one of the central issues that is the right to know one’s own origin, which would include ethnic, family, cultural, and genetic origin. Through the Declaration of the Rights of the Child of 1959 (Principle 3) (United Nations, 1959) and the International Covenant on Civil and Political Rights of 1966 (Article 24) (United Nations, 1966), the right of every child to registration, name and nationality was recognized. Although in those instruments it was not called “identity,” the Convention on the Rights of the Child (United Nations, 1989) incorporated such concept by establishing that the elements of identity are the birth rights to registration, nationality, name, and preservation of family relationships (Articles 7 and 8). Further on, Article 29(c) recognizes the importance of preserving the language and values which build cultural identity, thereby adding another element to a person’s right to identity. These elements can also be seen listed in Article 3 of the International Declaration on Human Genetic Data (UNESCO, 2003), which recognizes that a person’s identity should not be reduced to genetic characteristics since it involves complex educational, environmental and personal factors, as well as emotional, social, spiritual and cultural bonds with others, and implies a dimension of freedom.

A clear example of the importance of knowing one’s identity and the affectation that a person can suffer as a consequence of not knowing their affiliation is the case of Jäggi v. Switzerland. In its 2006 judgment, the European Court of Human Rights (ECHR) acknowledged the violation of Article 8 of the European Convention on Human Rights (right to respect for one’s private life) because it had been impossible for the applicant to obtain a DNA analysis of the mortal remains of his putative biological father.

The applicant in this case, Andreas Jäggi, a Swiss national, was born in 1939 (European Court of Human Rights, 2006a). Shortly before the applicant’s birth, a State-appointed adviser brought an action against A.H., his putative father, seeking a declaration of paternity and the payment of a contribution towards his maintenance, which he denied. When registering the applicant’s birth, his mother declared that his father was A.H. and gave him up for adoption. Mother and son met years later, and she informed him that A.H. was his father. Jäggi asserted that he had had regular contacts with A.H., but his alleged father always refused to undergo tests to establish his paternity until his death. In 1997 and 1999, Jäggi requested a DNA test on the mortal remains of A.H., but his application was refused by the trial courts. The Federal Court dismissed an appeal by the applicant on the ground that, at the age of 60, he had been able to develop his personality even in the absence of certainty as to the identity of his biological father. The ECHR took into consideration two special circumstances: the assessment that the rights of the deceased person in terms of respect for his body were not affected and that the lease for the grave was going to expire shortly. The family had no intention of renewing the lease, so the body of A.H. would be exhumed, a situation that would make it easier for Jäggi to take the sample.

The ECHR considered that individuals trying to establish their ancestry had a vital interest, protected by the Convention, in obtaining the information they needed to discover the truth about an important aspect of their personal identity. In the same line, the Court considered that an individual’s interest in discovering their parentage did not disappear with age; in fact, the opposite is true. Moreover, the applicant had always shown a real interest in discovering his father’s identity throughout his life. Such conduct implied moral and mental suffering, even though this had not been medically attested. That being the case, the ECHR considered that Switzerland had not secured to Jäggi the right to respect for his private life and held that there had been a violation of Article 8 (European Court of Human Rights, 2006b).

In another recently released case, it was learned that, in 2019, a 39-year-old man discovered, through an ancestry test, that he had a match with another half-brother. His parents had seen a fertility specialist in 1982 because the father had limited fertility, so the doctor suggested artificial insemination with the husband’s own sperm; he never told them about a sperm donor. The mother became pregnant and gave birth to the applicant in 1983. The man, upon contacting his brother and realizing they had both been conceived by artificial insemination in the same place where their respective families had been treated, sued the doctor who worked at the AZ Sint-Jan hospital before a court in Bruges.

In the first instance, the court declared that it had not been proven that the doctor manipulated the semen and that it could not be ruled out that the applicant was born from an adulterous relationship. On appeal, however, the court took a different position as there is no evidence that the mother met the biological father, had a relationship with him, or obtained his semen for insemination. The donor died, so his testimony could not be obtained. It was recognized that the semen was exchanged or mixed with donor semen without the knowledge of the parents, due to the negligence of the doctor, who, although he continued to deny the facts, had already withdrawn from practice. The court, by means of a judgment handed down on 22 December 2022, awarded 2,500 euros for non-material damage to the donor-conceived applicant since he suddenly discovered that his legal father was not his biological one. To date, the plaintiff has found seven siblings (Franck, 2023).

In this case, it was not mentioned that the treating physician is the biological father. Even so, it was shown that it was fraud with or without intent, which affected the image and self-perception of the plaintiff since he pointed out that, for years, he had doubts about his origin seeing that he did not resemble his father and sisters. He added that the doctor’s deception deprived him of the possibility of knowing his biological father given that, until the fraudulent behavior was discovered, he did not know about the existence of this parent who is impossible to contact because he is now dead.

In this sense, if the person has the right to preserve their identity, they therefore have the right to know the origin of each element that constitutes it. Even Article 7 of the aforementioned Convention proclaims the right to know and be cared for by one’s parents as far as possible. For a vast majority of people, whether they are children or adults, knowing the biological dimension of their identity is essential for their image, self-perception, and self-esteem, as it was clear in Jäggi’s case. Added to this comes the fact that, in the era of personalized medicine, knowing one’s genetic information acquires special relevance for health decision-making.

## 3 Discussion

In all cases, the doctors committed these acts deliberately because, even though there were no specific laws in the states or countries where the crimes were committed, they knew they were breaching their duty to their patients. Proof of this is that none of them openly offered their own semen to the women, and they decided instead to cover up the deception by offering supposedly donated sperm. In this light, the questionable argument that they did it to help their patients falls apart in the absence of clarity about the origin of the biological material. They were aware of the premeditated deception since they systematically lied about having a database of anonymous donors that they could not prove they had.

On social networks, some people, through their comments on related videos or reports about the fertility fraud scandals, excuse these doctors pointing out that, in the end, the women who were looking for an anonymous donor found one and were able to have a child. [For example, comments section of this video (VICE News, 2022)] The anonymity of the donors does not justify the lack of transparency on the part of the doctor towards their patients; for this reason, the patients must be informed about the main characteristics of the donor: ethnic group, age, and even social information, such as profession, occupation, or lack of criminal record. In some clinics, donors are asked to report genetic conditions and previous diseases. Although this last statement seems to refer to a quality standard, the problem is that, as already mentioned, not all countries have specific legislation, therefore there are legal gaps and dissimilar standards regarding the genetic examination of gamete donors.

Added to this, the growing practice of finding anonymous donors through social networks is of concern. The issue of informal donations is becoming a public-health problem given that, on the one hand, there is no record of the donors in the health systems and, thus they escape the regulation of their own countries and, consequently there is no control over the conditions in which reproductive services are given, neither over the tests that should be carried out on biological material. On the other hand, these donors are prolific because their sperm is used on multiple occasions exceeding the limit that is considered good practice in adequately regulated clinics. This escalates the risk of accidental “incest” between half-siblings or even between donors and donor-conceived individuals (Shepherd, 2023), increasing exponentially the genetic risks of conceiving a child with a close relative.

The public opinions on social media about fertility fraud also reveal a trivialization of paternity as if the only important thing was to obtain the biological material. This perspective ignores important issues, such as there has been a conception without consent since all the information requirements for decision-making are not met. On another note, the fact that unknown siblings may meet in small communities or cities, form a relationship, and possibly marry and have kids is not only against good practices in assisted reproductive medicine but also socially disturbing. This matter raises concerns about donor-conceived people who find out they have dozens of half-siblings, and maybe their known parents are not theirs. This incidental finding is a life-changing moment that can topple one’s notion of identity. A curious fact that draws attention to this is that Cline called his biological daughter, Jacoba Ballard, to tell her that her digging up the past was destroying his marriage because his wife considered his actions adultery (Zhang, 2019a). This reveals that, for many people, the provision of biological material is not an act indifferent to the social meaning of paternity, genetic inheritance, marital relations, and the notion of family.

As Adrian and contributors stated, anonymity can no longer be guaranteed for future and past donors given the availability of low-cost DNA testing and commercial international DNA ancestry sites (Adrian et al., 2022). Although the clinical significance of the results obtained from this type of tests is not fully validated since these sites are still refining their algorithms, the repercussion of the ancestry and paternity results provided by these companies is irrefutable.

Through DTC genetic testing, donor-conceived people may learn of their conception as a DNA surprise, an unexpected finding arising from a curiosity about one’s ancestry and/or genetic constitution. Similarly, both donors’ and recipients’ relatives can also face DNA surprises if the donor kept their status a secret or if the recipient had not disclosed their usage of assisted-reproduction services. For example, while the mothers in most of the above-narrated cases are certain of their maternity, many of the fathers who believed they were the biological fathers of their children found out the truth about their paternity through these DTC tests.

In all the fertility fraud cases, the whole family receives the impact when they learn about the manipulation of which they were victims. This bitter truth sneaks into family relationships and bashes the core of meanings, concepts, beliefs, and projects on which those families were built.

In countries that do not have a specific regulation on assisted reproductive medicine or countries that only regulate them technically and administratively, the challenge will be not only to legislate on fertility fraud but also to establish control mechanisms for clinics, supervise health staff and combat impunity for this crime. It would be desirable for the staff to be trained in both technical matters and the ethics of the doctor-patient relationship and bioethics.

Fertility fraud committed by any member of the medical staff must be considered as sexual assault, according to Madeira. The heart of it is a doctor betraying not only the trust but also the doctor-patient fiduciary relationship, literally inserting himself or some part of himself into the woman’s bodily cavity, betraying her autonomy, and adding his own genetic lineage into her family tree against her will. It is against her will because she was never given an opportunity to consent to it (Ettachfini).

In the United States, this reality has begun to be recognized. There are 17 states with fertility fraud laws or pending bills. The states of Arkansas (2021), Arizona (2021), Colorado (2020), Florida (2020), Indiana (2019), Iowa (2022), Kentucky (2022), Texas (2019), and Utah (2021) have already enacted legislation on this matter. Much of this regulation focuses on fraud committed by the physician (Right to Know, 2023). The task ahead is also to legislate on other cases of deception where fertility fraud is committed by someone other than health personnel.

The state of California and countries like Mexico illustrate another aspect that needs to be addressed about consent. In its Penal Code, California created the crime of using reproductive material other than that indicated by the donor’s consent form (except for sperm donors) and to implant reproductive material without the written consent of the recipient (California State Legislature, 2011). In Mexico, which, as mentioned, does not have specific regulations on assisted reproductive techniques, the General Health Law provides for a prison sentence for those who inseminate a woman without her consent, the duration of which depends on whether the pregnancy occurs or not [Article 466] (Cámara de Diputados, 1984).

This concrete aspect is surely regulated in various other countries either in the civil, health, or criminal spheres. That said, it is still necessary to update this type of provisions to contemplate the case of fertility fraud in which having given consent for insemination does not mean having consented not to be informed about the origin of the gametes.

Fertility fraud, however, is not the only practice that has eluded liability (Fox, 2022). There are distinct categories of reproductive misconduct that have been neglected, such as imposed conception, denied access to contraception, and forced sterilization. Lawmakers, judges and policymakers must tackle the different problems that have arisen in each of these categories in order to achieve a comprehensive and non-fragmented approach, for this insufficient revision of the corresponding regulations and principles has generated legal loopholes and inequality gaps.

Most of the past and present norms on assisted reproductive techniques and on anonymity focus on the reproductive rights of the parties involved or on the donor’s right to privacy, but, not always on the best interests of the child. This complex matter has many layers that need to be discussed in various public debates. Reproductive rights as any other right, should not be absolute, their exercise depends on the parents’ and on the child’s welfare, for example, just as it would be horrifying to defend the “right” of a man to rape his wife to get her pregnant, it is also horrible to excuse fertility fraud. The indignation generated by this example demonstrates that reproductive rights must be exercised with full consent, balancing the desires, needs and life projects of each one of those involved.

In the case of children, their rights and needs must always be prioritized over the interests of adults. In the care of children according to the Convention on Rights of the Child, States shall take all appropriate legislative and administrative measures (Article 3) to ensure that foster placement, shelter, refuge, and the system of adoption shall ensure the child’s wellbeing (Articles 20–22).

In many adoption systems, state authorities go to considerable lengths to find a family fit for the child and implement actions to preserve his/her safety such as, not turning the child over to a known sex offender or a person who has committed another type of serious crime. But when it comes to assisted reproduction, reproductive rights are taken into consideration with paramount importance, for example, in the cases of Dickson v. United Kingdom (Howie, 2007) or Yigal Amir in Israel (Nicholl, 2006), the judges considered that the prisoners convicted of murder in both cases, could resort to assisted reproduction, an issue that would not happen in adoption. I mention these extreme cases to highlight that the rights of the donor-conceived sometimes are not taken into consideration. It is important to note that gamete donation itself is not causing direct problems in the child; reckless practices around the assisted reproductive care are those that endanger the child’s welfare, for instance, (i) when the adequate examination of the biological material is not carried out, (ii) when an anonymous donor is contacted through social networks ignoring his/her background, giving a blind leap of faith for a stranger who could commit fraud against the gamete applicants, (iii) when clinics do not have a reliable database (as in the cases of fraudulent doctors mentioned above), (iv) when donor’s anonymity is priority over right to know and, (v) when assisted reproduction clinics do not have a rigorous procedure. In these reckless practices, rights to identity, full consent and to know one’s origin are not contemplated.

The donation of cells or organs is confidential when they are donated to a bank or go through the waiting list for transplants. Originally, given the novelty of the subject in the 80s, the regulation of gametes was similar. But nowadays, questions have arisen about the use of gametes, distinct than about organ or tissue donation. In relation to preserving the anonymity of the donor, the diverse legal criteria of the member countries of the European Union and/or of the Council of Europe draw attention. There have been various social changes since assisted reproductive techniques began to be regulated up to the present, but there is no uniformity in the moment and the way in which the new social demands are incorporated into public regulations and policies. It is difficult to venture a hypothesis about these differences, but a plausible explanation could be to avoid demotivating gamete donors and therefore, the risk of diminish the available biological material. As stated by Riaño et al., we cannot ignore the fact that there are possible conflicts of interest between economic factors and those related to the protection of the best interest of the child. The different requirements in the Americas and Asia are also considerable. The reason for not disclosing the information may also be related to the paternalism that still prevails in some societies. Just as the origin of an adopted child was hidden, some social customs also favor secrecy over the right to know one’s own origin.

In the era of globalization, another aspect that has been under discussion for many years is medical tourism, which is often generated by the absence or laxity of regulation in the destination countries. As Charo argues, medical tourism may simply be the search for a standard therapy at lower cost or with a shorter waiting period, but it may also mean seeking unapproved interventions available in countries with weak or nonexistent regulation (Charo, 2016). This puts users of health-service at risk, as they may be subject to techniques with poor quality control or there may be problems with the countries of origin, such as paternity acknowledgment in countries where gestational surrogacy is prohibited.

There is still a long way to go and pending heated debates to be had for balancing all interests of the parties involved in DTC tests. May points out that, concerning DTC genetic testing, the right to privacy and the misuse of genetic information are a problem because the lack of control has permitted a lawless environment; for example, reporters have shown how easy it is to send someone else’s sample for testing and receive a full report on that person (May 2018).

Concerns about consumers’ consent must also be addressed in both the public and academic debates. In the platforms of the provider companies, consumers consent to the use of their genetic information to obtain their diagnostic or ancestry results, but, in the past, they were unaware that their information could be compared with criminal databases (The Biometrics and Forensics Ethics Group, 2020). The Golden State Killer case is a paradigmatic example of a genetic-information usage not foreseen at the start of these companies. In 2018, the police sent the genetic information obtained from the biological material recollected in the crime scenes back in the 1970s to GEDmatch, and it was matched to relatives of the perpetrator, later identified as Joseph DeAngelo (Maher, 2018). The importance of the use that was given to the information is not questioned, since arresting a criminal is what the victims, their families, and society want, nor is the growing usefulness of DTC tests as a powerful tool. The point of the ethical debate regarding DTC testing is consent and the consumers’ right to be informed.

## 4 Conclusion

The claims made in this article do not yield conclusive results. They are instead clues intended to be useful in charting a path towards laws that address the problems that technological progress is uncovering.

In many nations, it is urgent to regulate assisted reproductive techniques. In countries that already have such regulation, it is crucial to condemn fertility fraud as a crime given how evident the attack against the integrity and autonomy of the person is. Furthermore, it is necessary to hold the physicians or any other type of perpetrator legally accountable.

The regulation to be proposed and discussed in the future, not only about fertility fraud but also regarding other reproductive misconducts, must weigh the rights of all those involved. That is, it should balance the rights of the donor-conceived people, the legal parents, and the donors who want to meet their children, e.g., to reveal a hereditary disease supervening on the donation.

Based on this right to know one’s origins and the overwhelming advance of DTC genetic testing, the anonymity of participants in assisted reproductive techniques (donors and recipients) becomes increasingly untenable. Anonymity, when combined with little precision in the control of processes in reproductive-medicine clinics or with nefarious intentions, such as those of the aforementioned doctors, has proven not to be the best solution for the interests of all those involved in assisted reproduction.

At present, the platforms provided by DTC genetic testing have many potential uses that must be examined through an ethical perspective.

The constant review of the informed-consent process as an unfinished questioning about good practices and ethical, legal, and social implications is essential in the genomic era.
